# From Association to Causality: Next-Generation GWAS, Pangenomes, and Multi-Omics Driving Gene Discovery and Precision Breeding in Cereal Crops

**DOI:** 10.3390/biology15181673

**Published:** 2026-09-21

**Authors:** Helmy M. Youssef, Radwa Y. Helmi, Andreas Börner, Mahmoud M. Sakr, Mohamed El-Soda

**Affiliations:** 1 Centre for Converging Science and Emerging Technology (CoSET), Benha National University, Al Obour 13518, Egypt; msakr@asrt.sci.eg; 2 Department of Botany, Faculty of Agriculture, Cairo University, Giza 12613, Egypt; 3 Chair of Plant Breeding, Institute of Agricultural and Nutritional Sciences, Martin Luther University Halle-Wittenberg, Betty-Heimann-Str. 3, D-06120 Halle (Saale), Germany; 4 Genetics and Cytology Department, Biotechnology Research Institute, National Research Centre, Giza 12622, Egypt; ry.esmail@nrc.sci.eg; 5 Leibniz Institute of Plant Genetics and Crop Plant Research (IPK), Corrensstr. 3, OT Gatersleben, D-06466 Seeland, Germany; boerner@ipk-gatersleben.de; 6 Plant Biotechnology Department, Biotechnology Research Institute, National Research Centre, Giza 12622, Egypt; 7 Department of Genetics, Faculty of Agriculture, Cairo University, Giza 12613, Egypt; mohamed.elsoda@agr.cu.edu.eg

**Keywords:** cereals, GWAS, gene identification, crop improvement, multi-omics, genomic selection, haplotype breeding, wheat, barley, maize, rice

## Abstract

Genome-wide association studies (GWASs) have become a central tool in plant genomics, providing high-resolution access to the genetic architecture of complex traits in cereal crops. This review observes the application of GWASs to biotic and abiotic stress responses and nutritional quality in rice (*Oryza sativa*), wheat (*Triticum aestivum*), maize (*Zea mays*), and barley (*Hordeum vulgare*). However, conventional SNP-based GWASs may overlook structural and presence–absence variation, and significant associations do not by themselves identify the causal gene or variant. Recent methodological developments, including multi-locus and environment-aware models, can improve the detection of complex genetic effects, while pan-genomic and graph-based references broaden the range of genomic variation available for association analysis. Integrating GWASs with transcriptomics, metabolomics, and proteomics provides additional functional evidence for candidate-gene prioritization and interpretation of associated loci. Fine mapping and functional validation, including genome-editing approaches, further help to test candidate-gene function. Together, these advances strengthen the progression from statistical association to biological interpretation and breeding-relevant genetic variation. This review discusses their current applications, limitations, and potential for improving cereal breeding.

## Simple Summary

Cereal crops such as rice, wheat, maize, and barley are fundamental to global food security, yet their production is increasingly challenged by climate change, emerging pests and diseases, and environmental stresses. Identifying the genetic basis of traits related to stress tolerance, disease resistance, and nutritional quality is therefore essential for accelerating crop improvement. Genome-wide association studies (GWASs) have contributed substantially to this goal by linking genomic variation with complex phenotypes. However, conventional GWASs often focus primarily on single-nucleotide polymorphisms and may overlook structural variation, while association signals alone do not always reveal the true causal genes. Recent advances in pangenomics, multi-omic integration, and gene-editing technologies are helping to overcome these limitations by improving candidate-gene identification and functional validation. Together, these approaches are moving cereal genomics from statistical association toward biologically validated targets for developing more resilient and nutritious crop varieties.

## 1. Introduction

Cereal crops such as wheat (*Triticum aestivum*), rice (*Oryza sativa*), maize (*Zea mays*), and barley (*Hordeum vulgare*) constitute the primary source of calories and proteins for more than half of the global population [1]. Ensuring their productivity, resilience, and nutritional quality under rapidly changing climatic conditions remains a major challenge for modern agriculture [2]. Understanding the genetic basis of complex agronomic traits in these crops is therefore a central objective of plant genomics and breeding [3,4]. Over the past two decades, Genome-Wide Association Studies (GWASs) have become a transformative approach for dissecting complex traits by exploiting natural genetic diversity present in large germplasm collections [5,6]. The practical application of GWASs on plants was strengthened by high-density genotyping and statistical models that account for population structure and kinship [7,8]. Initial demonstrations based on Arabidopsis thaliana provided proof that historical recombination in natural populations can be used to map loci at much higher resolutions than traditional biparental quantitative trait locus (QTL) mapping [7]. This concept rapidly translated to cereals through the establishment of large diversity and association resources, including the 3000 Rice Genomes Project, the maize association and Nested Association Mapping (NAM) populations, and the CIMMYT Wheat Association Mapping Initiative (WAMI) panel [9,10,11,12].

Unlike linkage mapping, which is limited by a few recombination events and narrow allelic diversity, GWASs reflect historical recombination accumulated over thousands of generations, allowing mapping at the near-gene resolution [13]. This advantage is particularly important for cereals, where diverse germplasm can reveal adaptive alleles for complex traits, including stress tolerance, as demonstrated by the introgression of wild barley alleles associated with improved salinity tolerance [14]. Early GWASs in rice demonstrated the power of this approach to identify loci controlling flowering time, grain width, and domestication traits [15]. Similar successes in maize and wheat further established GWASs as a central tool in cereal genomics [16,17]. Advances in high-density genotyping, including SNP arrays and genotyping-by-sequencing (GBS), have increased marker density and genome coverage, thereby improving GWAS resolution and power [18,19]. Concurrently, statistical innovations such as mixed linear models (MLMs), the three-variance-component multi-locus random-SNP-effect mixed linear model (3VmrMLM), FarmCPU, and BLINK have addressed the confounding effects of population structure and reduced false positives [8,20,21,22]. These methodological developments transformed GWASs from an exploratory mapping tool into a strong framework routinely applied across cereals.

In recent years, GWASs have expanded beyond SNP–trait associations. Recognizing that single reference genomes do not capture the full genetic diversity of cereals has driven the development of pan-genomes and graph-based resources that incorporate non-reference and structural variation [23,24]. Pan-genomic resources expand the catalog of non-reference and structural variation available for association analysis, particularly in structurally complex cereal genomes such as wheat and barley [23,25]. Integrating GWASs with functional genomics and post-GWAS analyses helps move from statistical associations toward the identification of candidate causal genes [26]. Transcriptome-wide association studies (TWASs), expression quantitative trait locus (eQTL) mapping and metabolite-based genome-wide association studies (mGWASs) link genetic variation to gene-expression or metabolite phenotypes, providing functional evidence for candidate-gene prioritization and pathway interpretation [26,27,28,29]. Genome editing tools such as CRISPR/Cas can provide direct functional tests of prioritized candidate genes through targeted knockout or allele modification [26,30].

The translational value of GWAS is increasingly evident in modern breeding. GWAS-derived markers are integrated into marker-assisted selection (MAS) pipelines, while whole-genome marker data are used in genomic selection (GS) models to predict breeding values [31,32]. Integration of high-throughput phenotyping and machine-learning approaches can further support genomic prediction and breeding decisions [33,34]. Despite its strengths, GWASs face challenges including limited power to detect rare alleles, linkage disequilibrium obscuring causal variants, and genotype-by-environment interactions that reduce the stability of detected effects [35,36,37]. Addressing these limitations requires appropriately designed populations, multi-environment phenotyping, and statistical approaches that explicitly account for environmental heterogeneity; meta-analysis can further combine association evidence across environments or studies [38,39].

Given the rapid evolution of GWAS methodologies and their expanding integration with pan-genomics, multi-omics, and breeding technologies, a synthesis is needed that connects methodological advances with the progression from association signals to candidate-gene validation and breeding application. This review therefore follows the progression from association discovery to candidate-gene prioritization, functional evidence and validation, and finally the use of validated loci in cereal breeding, while examining the methodological and genomic approaches that support each step.

## 2. The Genome-Wide Association Study (GWAS) Revolution

Building on the historical trajectory outlined above, this section sets out the conceptual and practical basis for the rest of the review. We first trace the development of crop GWASs from early association mapping to current next-generation approaches (Section 2.1), and then summarize the specific ways in which GWASs contribute to crop development (Section 2.2).

### 2.1. From Early Association Mapping to Next-Generation GWASs: Timeline and Milestones

**Figure 1:**
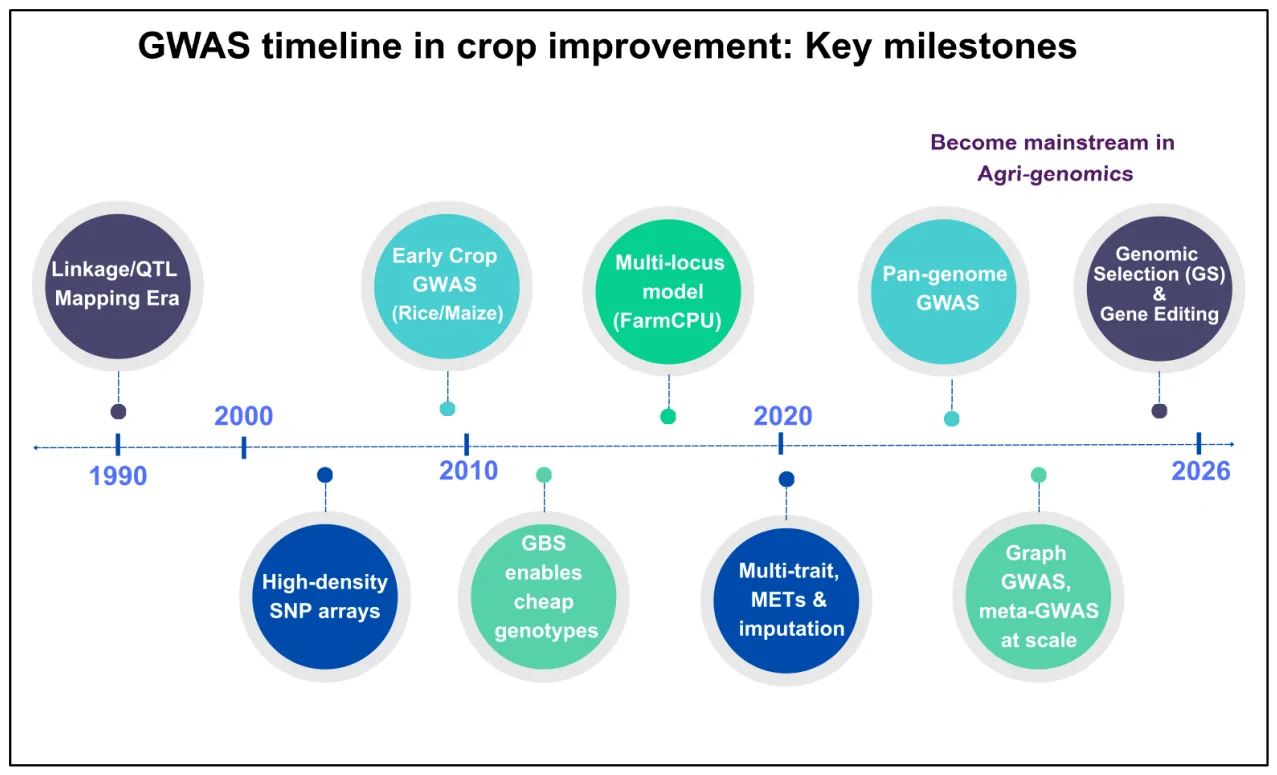
Timeline of GWAS in crop improvement: selected milestones from early association mapping to next-generation approaches and deployment. Key transitions for breeding translation include improved control of confounders through mixed models, affordable dense genotyping (GBS/whole-genome resequencing (WGS)), multi-locus and multi-environment methods, and structural variant (SV)-aware pangenome and graph frameworks integrated with phenomics, genomic selection, and genome editing.

Genome-wide association studies (GWASs) in crops emerged from a broader transition in quantitative genetics: from family-based linkage/QTL mapping to population-based association mapping enabled by dense genotyping and improved statistical control of relatedness [8,13]. Crop GWASs have since progressed from SNP-array and single-reference designs through genotyping-by-sequencing and whole-genome resequencing to current multi-locus and multi-environment approaches that incorporate structural variants, pan-genome and graph references, high-throughput phenotyping, genomic selection, and genome editing (Figure 1) [40,41,42].

### 2.2. Why GWASs Matter for Crop Development

**Figure 2:**
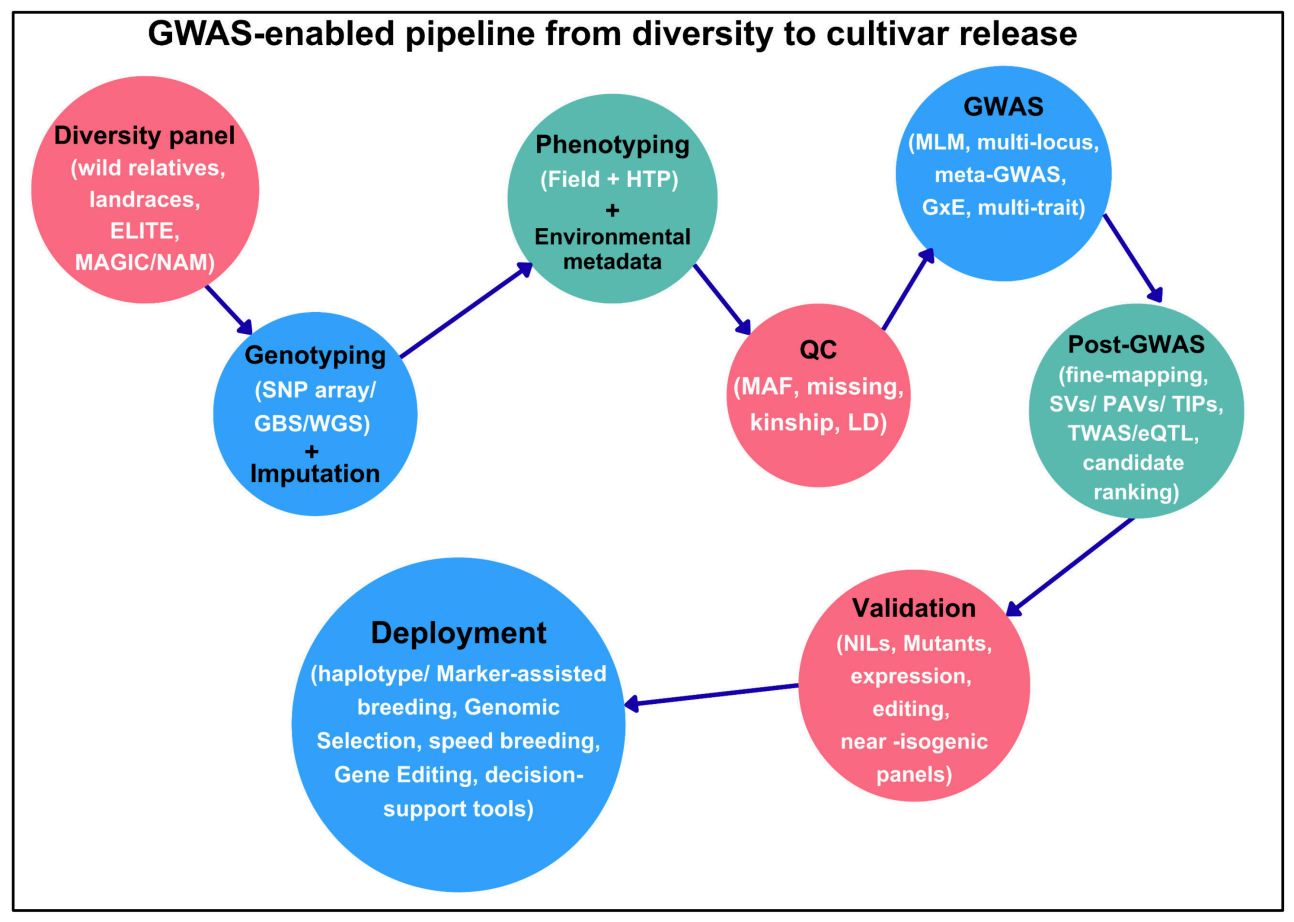
A GWAS-enabled breeding pipeline from genetic diversity to cultivar release. The schematic follows the sequence from germplasm assembly and phenotyping, and genotyping and association analysis, to candidate-gene validation and deployment via marker-assisted selection, genomic selection, and genome editing, indicating where design decisions influence discovery power and translational value.

GWASs contribute to crop development through three complementary routes. First, they provide a discovery layer: pinpointing genomic region controlling agronomic and adaptive traits, including alleles hidden in landraces or wild relatives [32]. Second, they support prioritization and decision-making: haplotype or marker assays developed from robust associations can accelerate selection, while integration with genomic selection (GS) can improve prediction when large-effect loci exist [31,43]. Third, GWASs can contribute to mechanistic interpretation when association signals are integrated with functional-genomic and multi-omic evidence, helping to prioritize candidate genes and biological pathways for further validation [26,44]. Integrative omics approaches are increasingly used to investigate drought responses in crops [45]. In wheat, recent work has combined GWAS, eQTL mapping, transcriptomics, and functional validation to dissect WUE and drought resilience [46]. In barley, pangenome work has revealed previously inaccessible variation at structurally complex loci, including disease resistance regions [47]. In maize, diverse panels and pangenomes support association mapping for drought resilience and developmental traits [48]. In rice, the accumulation of multiple GWAS datasets has enabled meta-analysis at a very large-scale using graph-based pangenome references, increasing power and discovery of novel QTL [49]. Figure 2 summarizes an end-to-end GWAS-enabled breeding pipeline, highlighting where design decisions influence discovery power and translational value.

## 3. Methodological Advances in GWAS

Genome-wide association studies (GWASs) are based on the principle that phenotypic variation in traits is associated with genetic variation at specific loci, particularly single-nucleotide polymorphisms (SNPs), across a diverse population [13]. Unlike traditional biparental mapping methods that limit genetic resolution due to reduced recombination events, GWASs exploit the historical recombination present in natural populations, allowing for finer mapping of trait-associated loci [13,36]. In plants, particularly cereals, GWASs have become a preferred approach for uncovering the genetic basis of complex, quantitative traits influenced by multiple genes and environmental interactions [39,50].

The process of GWASs begins with the assembly of a diverse and representative panel of genotypes. These genotypes are then phenotyped for traits of interest under various conditions and genotyped using high-density molecular markers, such as SNP arrays or next-generation sequencing (NGS)-based genotyping-by-sequencing (GBS) [19,51]. Statistical models are applied to test for associations between markers and phenotypes, taking into account factors such as population structure and kinship to reduce false-positive associations. The mixed linear model (MLM) is among the most commonly used statistical frameworks, although newer models like FarmCPU and BLINK offer enhanced power and computational efficiency [8,20,22].

### 3.1. Population Choice and Sampling Strategy

Panels used for crop GWAS fall into three broad classes [39]: (1) Diversity panels (landraces, historical cultivars, and wild relatives) maximize allelic diversity but usually have strong population structure and variable LD [52,53]. (2) Elite breeding panels (advanced lines, regional breeding programs) offer direct relevance to selection targets and usually reduced structure, but capture less rare diversity [16]. (3) Structured multi-parent populations: Multi-parent Advanced Generation Inter-Cross (MAGIC) and NAM combine balanced recombination with controlled structure and enable both mapping and prediction [11,54]. For wheat and barley, selfing increases LD, which boosts power for detecting QTL but reduces mapping resolution; thus, larger panels and high marker density are needed for fine-mapping [55]. In maize, rapid LD decay and strong haplotype diversity often deliver higher resolution but require dense markers and careful control of relatedness; NAM designs are particularly powerful [56]. In rice, panels can be designed within subspecies groups (indica, japonica) to reduce confounding while enabling within-group discovery; large-scale meta-analysis across panels further increases power [15,57].

### 3.2. Phenotyping and Environmental Metadata

In crop breeding, the phenotype is rarely a single value; it is a function of genotype, management and environment [33]. Multi-location and multi-year trials introduce spatial heterogeneity and genotype-by-environment interaction (G×E) that can mask or distort marker–trait associations if not properly modeled. Robust GWASs, therefore, depend on phenotypic data that are accurate, repeatable, and accompanied by structured environmental metadata [33,58]. Best-practice phenotyping pipelines apply experimental designs (e.g., augmented or alpha-lattice layouts) with replication and spatial correction, and derive best linear unbiased estimates (BLUEs) or predictors (BLUPs) to summarize genotype performance across environments [59,60]. High-throughput phenotyping (HTP) platforms, using RGB, multispectral, hyperspectral, thermal, and LiDAR sensors, now capture trait dynamics non-destructively across the growing season, converting single-endpoint measurements into time-resolved trajectories [33]. Equally important is systematically recording environmental covariates (soil properties, weather, and management) as standardized metadata, since these enable envirotyping and the explicit modeling of G×E that underpins environment-aware and multi-environment GWASs [58,61,62].

### 3.3. Genotyping: Arrays, GBSs, WGS and Imputation

Marker density and variant spectrum jointly determine statistical power and the ability to identify causal variation [19]. Arrays provide standardized SNP sets and are cost-effective for large wheat and barley panels, but they can bias discovery toward common alleles in the array design [63]. GBSs provide broader genome coverage at a lower cost but introduces missingness, which must be addressed through imputation and stringent QC [64,65]. Whole-genome resequencing (WGS) is increasingly feasible for rice and maize and enables detection of rare variants and non-SNP classes of SV, including presence–absence variation (PAV) and transposable element insertion polymorphism (TIP) [12,66]. Imputation strategies using reference haplotype panels can increase marker density and harmonize datasets across platforms, enabling meta-GWASs and cross-study comparability [67]. In polyploid wheat, the complexity of homoeologous genomes complicates genotype calling and subgenome-specific variant assignment, while pangenome resources can help reduce single-reference bias [25]. Reference-free k-mer-based GWASs offer a complementary route for genotyping platforms: by testing short-sequence k-mers directly against the trait rather than pre-called variants, they capture SNPs, indels, and structural variation simultaneously and work even without a complete reference assembly, at the cost of heavier computation and less mature analysis tools [68,69].

### 3.4. Statistical Models and Computational Methods

The core problem in studies is separating true genotype–phenotype associations from confounders due to relatedness, population structure, and environment [70]. Crop panels often show both deep ancestral structure and recent breeding relatedness [36]. Mixed linear models (MLMs) address these issues by incorporating population structure covariates (e.g., principal components) and a random effect capturing kinship [71]. However, single-locus MLMs can be conservative for polygenic traits, and LD can spread association signals across wide intervals. Thus, many studies now employ multi-locus approaches, multi-trait models, and meta-analysis to increase power and biological interpretability [39].

The statistical methodology underpinning GWASs has evolved considerably to address challenges, including population structure, kinship, gene-by-environment interactions (GEIs), and computational scalability for increasingly large datasets [72]. Mixed linear models (MLMs) remain fundamental, accounting for population structure through fixed effects and kinship through random effects. Recent innovations include the three-variance-component multi-locus random-SNP-effect mixed linear model (3VmrMLM), which can detect quantitative trait nucleotides (QTNs) as well as QTN-by-environment and QTN-by-QTN interactions in GWASs [21].

### 3.5. Mixed Models and Multi-Locus Approaches

Multi-locus methods, including FarmCPU, BLINK, and the mrMLM family, provide alternative strategies for detecting multiple loci and can improve power when single-locus models become overly conservative for complex traits [20,22,73]. Bayesian sparse linear mixed models provide an alternative framework for traits influenced by both sparse large-effect loci and a polygenic background [74]. In practice, a useful strategy is to run a conservative MLM to establish high-confidence signals and complement it with a multi-locus method to detect additional loci, and then prioritize loci that replicate across environments, populations, or methods [75].

### 3.6. Multi-Trait, Time-Series, and Environment-Aware GWASs

Because many agronomic traits are genetically correlated, including grain-size components, flowering-related traits, and drought-response indices, multi-trait GWASs can exploit these correlations to increase statistical power and help distinguish pleiotropy from close linkage [16,76]. For HTP-derived time-series traits, functional GWAS models treat trajectories as phenotypes, enabling mapping of growth stages or stress-response dynamics [77]. Environment-aware GWASs incorporate environmental covariates and test marker-by-environment interactions, aligning discovery with the target population of environments [37]. These approaches are particularly relevant for drought and heat tolerance in wheat and maize, where performance depends strongly on seasonal patterns.

### 3.7. Meta-GWAS and Cross-Study Integration

**Figure 3:**
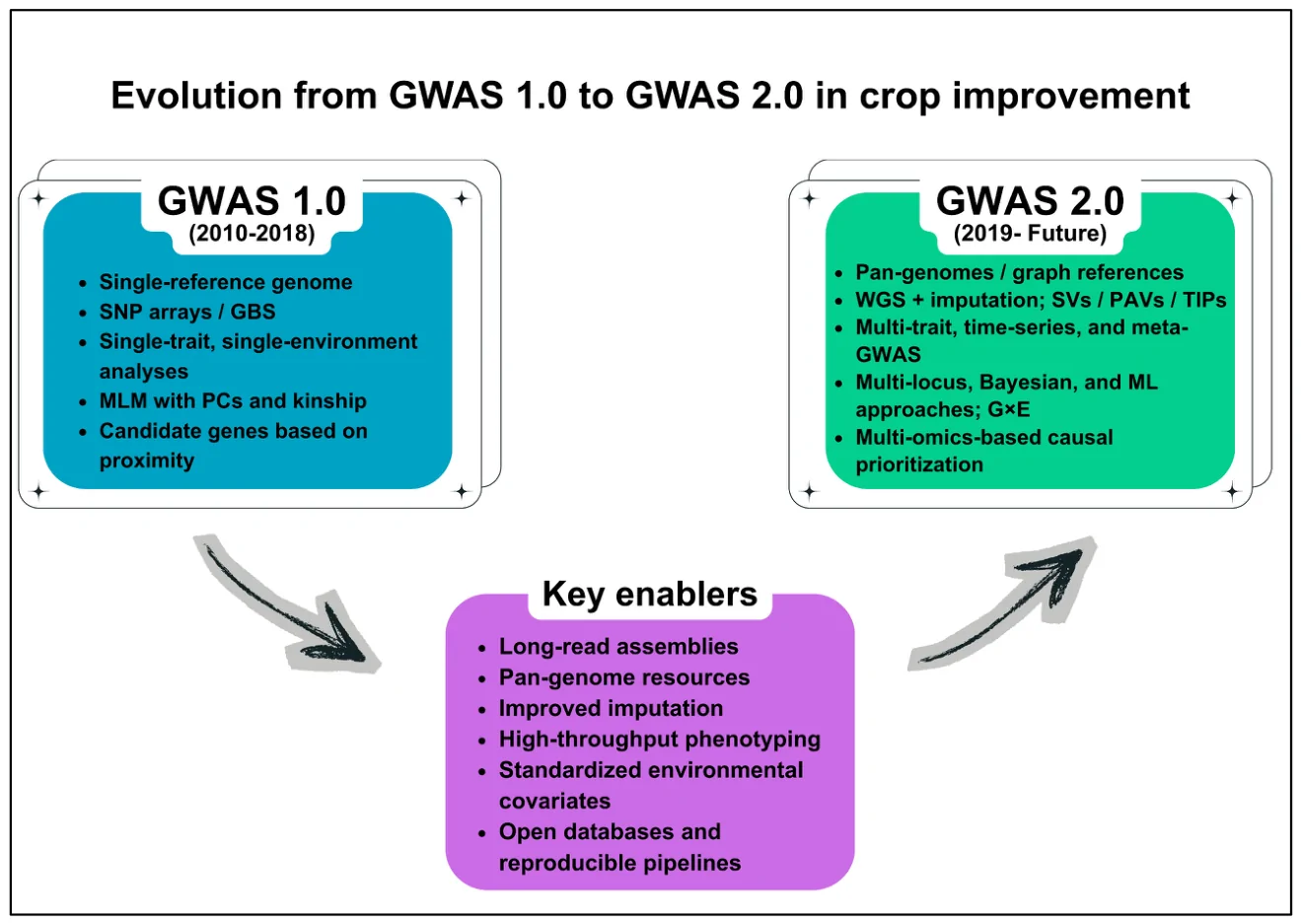
Evolution from GWAS 1.0 to GWAS 2.0 in crop improvement. Comparison of first-generation GWASs (single-locus mixed models, SNP arrays, single-reference genomes) with current approaches that combine multi-locus and multi-environment models, whole-genome and structural-variant data, pan-genome and graph references, and integration with high-throughput phenotyping, genomic selection, and genome editing.

Meta-analysis can improve discovery by combining association evidence across independent studies or environments and reducing study-specific noise [38]. Critical requirements include harmonized phenotype definitions, consistent QC and imputation, and explicit modeling of heterogeneity across studies. The methodological transition from single-locus, single-reference GWASs toward multi-locus, multi-environment, and SV-aware approaches is summarized in Figure 3.

## 4. Beyond SNPs: Pangenomes, Structural Variation, and Graph References

The methodological advances described so far operate largely within a single-reference framework. A complementary line of progress concerns the reference itself: the shift from single reference genomes to pan-genomic resources represents one of the most significant recent advances in crop genomics [78,79]. A pan-genome includes all genomic sequences present across a species, including core genes shared by all individuals and variable genes present in only some accessions [40]. This broader representation overcomes reference bias, a critical limitation where mapping short reads to a single reference genome fails to detect sequences absent from that reference [80].

Graph-based pan-genomes represent genomic diversity as networks rather than linear sequences, with sequence variants depicted as alternative paths through the graph [81]. This architecture enables more accurate read mapping in highly divergent regions and comprehensive detection of structural variants (SVs), including insertions, deletions, inversions, and presence-absence variations (PAVs). Recent studies have constructed graph pan-genomes for major crops, including rice and wheat [49,82]. Pan-genome integration has had a clear impact on GWAS power. In rice, a meta-analysis of 7765 accessions using a graph-based pan-genome derived from 356 rice accessions identified 6,604,898 SNPs and 42,879 PAVs [49].

**Figure 4:**
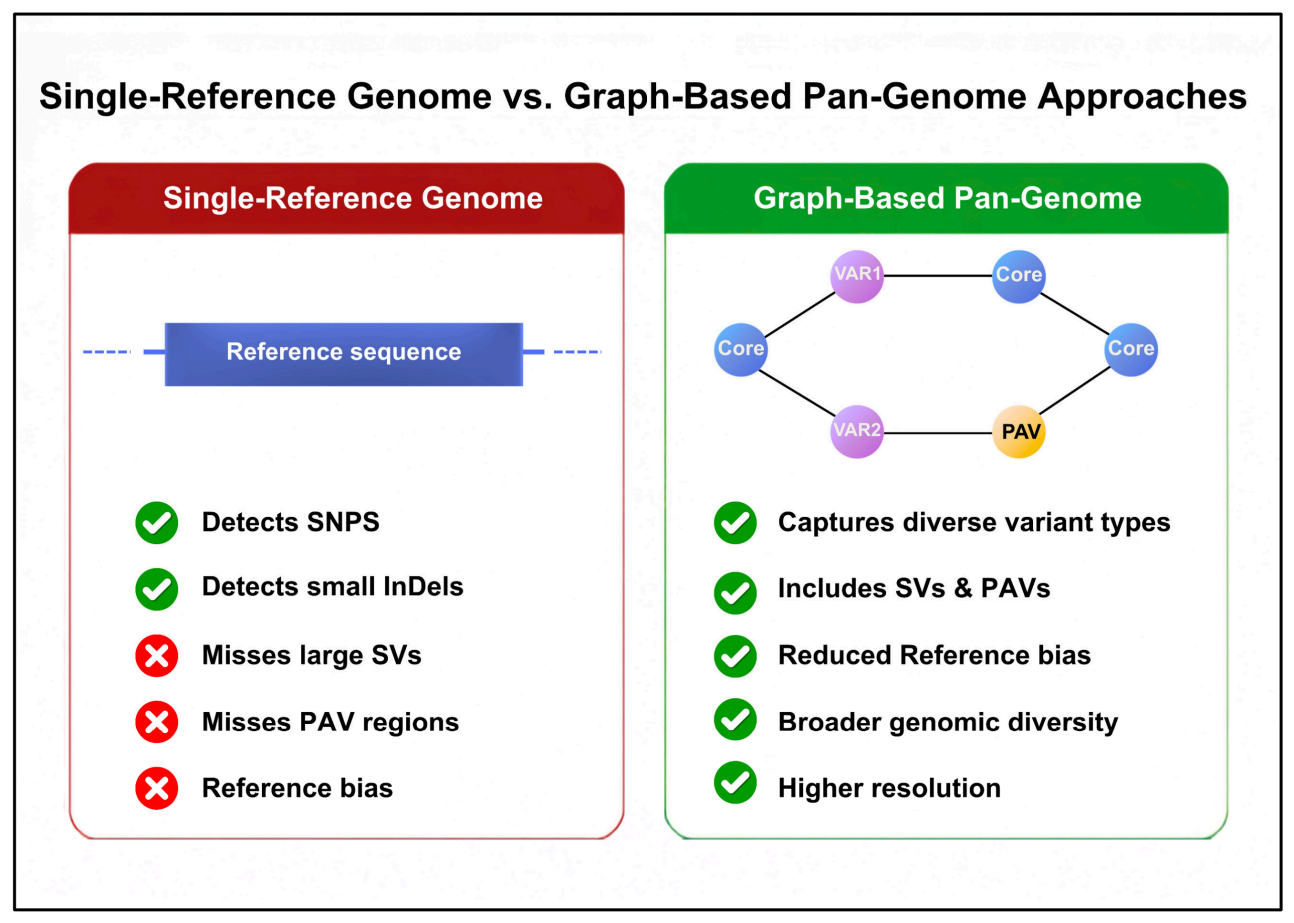
Single-reference versus pan-genome approaches to GWASs. Schematic contrasting read mapping and variant detection against a single linear reference genome (**left**) with a graph-based pan-genome that incorporates multiple assemblies (**right**), illustrating how presence/absence variation, structural variants, and non-reference sequence become accessible to association analysis under the pan-genome model.

This pan-genome-based meta-GWAS improved QTL detection by up to 43% and recovered up to 37.88% more heritability than individual GWAS analyses; two novel QTLs governing grain width and grain length were subsequently validated through CRISPR/Cas9 [49]. The conceptual difference between single-reference and pan-genome–based mapping is illustrated in Figure 4.

Reference bias is a major limitation of GWASs in crops: variants absent from the reference cannot be directly genotyped or mapped, and read mapping errors around repetitive regions can reduce power and inflate false signals [83]. Pangenomes address this by representing multiple high-quality assemblies, capturing gene presence/absence, copy-number variation, large insertions/deletions, and transposable element polymorphisms [79]. A barley pangenome study demonstrated extensive structural variation in wild and domesticated barley, including variation at complex loci associated with adaptation and disease resistance that became accessible through long-read assembly [47]. In maize, a pangenome built from drought-contrasting germplasms identified regulatory diversity and candidate genes enhancing drought resistance [48]. In rice, graph-based pangenome mapping enables inclusion of PAVs and other non-reference variants, and a meta-GWAS using a graph-based pangenome increased gene-mining efficiency for key agronomic traits [49]. Similarly, a transposable element insertion polymorphism (TIP)-GWAS using a rice pangenome graph identified cold-tolerance genes and provided a web resource to explore polymorphic TEs [84]. Collectively, these studies indicate that future crop GWASs will be increasingly ‘pangenome-aware’, especially for traits linked to structural variation [83].

### Practical Implications for Wheat, Barley, Maize, and Rice

This subsection focuses on how pangenome resources can improve variant discovery and association analysis in the four major cereals, whereas Section 8 discusses trait-specific GWAS findings and their breeding relevance. For wheat, polyploidy and a large repetitive genome make reference bias particularly severe; expanding wheat pangenomes and improving graph alignment will help capture adaptive variation from landraces and wild relatives [85,86]. For barley, pangenome resources reveal structural variation at complex loci associated with disease resistance, domestication-related architecture, and malting traits [47]. For maize, pangenome resources capture extensive gene presence/absence and regulatory structural variation that a single reference genome may miss [87,88]. For rice, expanding pangenome resources provide a broader representation of structural and non-reference variation that can support association analyses and future breeding applications [89].

## 5. From Association to Causality: Post-GWAS Fine Mapping and Functional Validation

Richer variant catalogs and pan-genomic references expand what GWASs can detect, but a significant association is only a starting point. Moving from association toward causality requires convergent evidence from fine mapping, functional genomics, cross-population replication, and experimental validation [26].

### 5.1. Fine Mapping and Haplotype-Based Resolution

Fine mapping aims to reduce the candidate interval to a small set of variants or genes [87]. Approaches include local LD analysis, conditional GWASs (testing peaks while controlling for lead variants), multi-locus modeling, and haplotype-based association. Haplotypes can capture the combined effect of multiple linked variants and can facilitate the development of breeding assays. Where possible, integration of recombination maps and local ancestry can sharpen intervals, particularly in elite panels with long LD. Multi-parent populations and high-recombination designs provide an additional path to resolution [54].

### 5.2. Integrating Transcriptomics and eQTL/TWAS Frameworks

Co-localization tests whether GWASs and eQTL signals are consistent with a shared causal variant, whereas summary-based Mendelian randomization (SMR) evaluates whether genetically regulated gene expression is associated with the trait. Section 6.1 details how eQTL and TWAS integration operates more broadly as a discovery layer across cereals.

### 5.3. Experimental Validation: From Mutants to Genome Editing

Validation options vary by crop and resource availability [90]. Near-isogenic lines (NILs) and introgression lines enable field-relevant validation in breeding backgrounds [91]. In barley, extensive collections of natural and induced mutants provide valuable resources for dissecting agronomic traits and experimentally validating prioritized candidate genes [92]. Once a candidate gene underlying such a mutant is identified, heterologous expression of the encoded protein provides an independent, biochemical layer of validation; for instance, the barley *Xantha-f*, *-g* and *-h* genes were expressed in a recombinant system to reconstitute magnesium chelatase activity, confirming the function assigned to the corresponding loci [93]. Genome editing approaches such as CRISPR/Cas enable direct functional testing of prioritized candidate genes through targeted knockout or allele modification [90]. For example, targeted knockout of the barley *Ycf54* gene generated an allelic series whose phenotypes demonstrated its essential role in chlorophyll biosynthesis, providing direct functional evidence for gene function [94]. In maize, integration of GWAS and transcriptome profiling identified *MADS26* as a candidate regulator of seed germination, with overexpression providing functional support for its role [95]. Validation of breeding relevance should extend to multi-environment field testing, because trait effects may vary across environmental conditions [33].

## 6. Multi-Omics Integration with GWASs

Having traced how pan-genomic references sharpen the association signal and how fine mapping narrows it to a short list of candidates, the next step toward causality is to layer independent molecular evidence onto that shortlist. Integrating GWASs with multi-omics technologies can improve candidate-gene prioritization and the biological interpretation of complex crop traits [96]. Multi-omics approaches, including transcriptomics, metabolomics, proteomics, and epigenomics, enable an integrated understanding of the molecular mechanisms underlying complex traits [44]. These layers of biological information, when aligned with GWAS results, can validate candidate genes, refine association signals, and reveal causal pathways that traditional GWAS alone might miss [97].

### 6.1. Transcriptomics and Expression QTLs

Each omics layer answers a different question about a GWAS signal. eQTL mapping asks whether genetic variants are associated with variation in gene expression, whereas TWAS tests whether genetically regulated gene expression is associated with the trait of interest [27,98]. Combining GWASs with transcriptome-wide association studies (TWASs) can improve candidate-gene prioritization by integrating genetic association with expression-based evidence [28,99,100].

In maize, association analyses using transcript abundance as the independent variable identified trait-associated transcripts for developmental traits, complementing SNP-based GWAS [101]. Transcript abundance can reflect the regulatory effects of genetic variation, including structural variants that influence gene expression [102]. In maize, high-throughput multiple optical phenotyping combined with GWAS identified 1529 QTLs and 2318 candidate genes associated with image-based drought-response traits (i-traits) [103].

Spatial transcriptomics, an emerging technology, offers exceptional resolution by mapping gene expression patterns within specific tissue regions [104]. Integrating spatial transcriptomics with additional omics layers can provide spatially resolved molecular information that helps characterize regulatory networks involved in plant development and stress responses [96,105].

### 6.2. Proteomics and Metabolomics in GWAS

Protein quantitative trait locus (pQTL) analysis investigates whether genetic variants are associated with variation in protein abundance, providing evidence at a molecular level downstream of gene expression [106]. Similarly, metabolite-based genome-wide association studies (mGWASs) address whether genetic variants influence measurable biochemical phenotypes, thereby establishing a direct link between genotype and metabolite profiles or quality-related traits that are ultimately relevant for crop improvement and breeding [107]. Whereas transcriptomics reports on gene expression, proteomics and metabolomics add further layers by capturing downstream molecular phenotypes closer to the ultimate trait of interest [108]. In maize, pQTL mapping using an integrated multi-omics approach identified candidate genes associated with kernel size, revealing roles for genes involved in signal transduction and amino acid metabolism [109]. Such pQTL evidence provides functional context for genomic variation and can support the prioritization of breeding-relevant candidate genes [110].

Metabolomics adds another critical layer, especially in dissecting nutritional traits. GWASs of biochemical and grain-quality phenotypes has identified loci controlling compounds such as tocochromanols in maize and β-glucan content in barley [111,112]. Metabolic profiles can also contribute to trait prediction in breeding populations; in maize, metabolic prediction showed substantial accuracy for complex heterotic traits [113].

Proteomics, while more challenging due to dynamic protein abundance and post-translational modifications, has also been applied to cereals to illuminate stress responses and developmental transitions [114]. Epigenomic studies, including chromatin accessibility and DNA methylation profiling, provide an additional layer for understanding gene regulation and stress responses in plants [115]. In maize, single-cell chromatin accessibility profiling identified cell-type-specific cis-regulatory elements enriched for phenotype-associated genetic variants, illustrating how epigenomic maps can help prioritize regulatory regions linked to complex traits [116].

Beyond metabolomics, ionomic GWASs can identify loci controlling elemental composition; in rice, a GWAS of 17 mineral elements in grain revealed the genetic architecture of ionomic variation [117]. In maize, a metabolome-based GWAS of kernel metabolites identified 1459 locus–trait associations, with eQTL and linkage evidence supporting many loci and mutant and transgenic analyses validating two candidate genes [118].

Integrating multiple omics layers requires computational frameworks capable of combining heterogeneous genomic, transcriptomic, proteomic, and metabolomic information for candidate-gene prioritization and biological interpretation [119,120].

### 6.3. Machine Learning for Multi-Omics Analysis

Machine learning and deep learning can integrate evidence across multiple omics layers to prioritize candidate genes and predict phenotypes that individual data layers may not resolve alone. A recent example is GP-WAITER, a hybrid convolutional-Transformer architecture that embeds GWAS-derived SNP weights into a self-attention model for genomic prediction; benchmarked across soybean, maize, rice, and wheat, it outperformed seven established genomic-prediction methods while remaining interpretable through SHAP-based attribution of individual variants [121].

**Figure 5:**
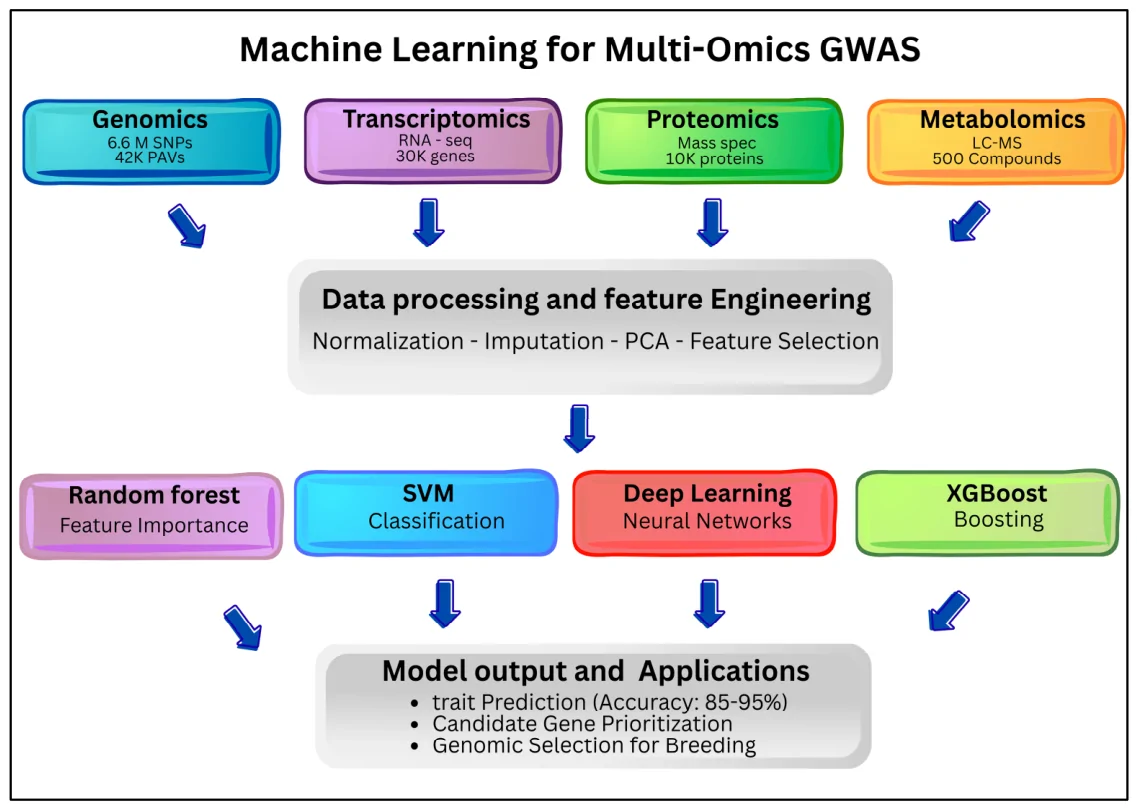
Machine learning for multi-omics GWASs. Schematic of how supervised and deep-learning models integrate genomic, transcriptomic, proteomic, and metabolomic layers to improve candidate-gene prioritization, capture non-linear and epistatic effects, and enhance trait prediction for genomic selection.

Machine learning (ML) and deep learning (DL) methods are increasingly used to analyze high-dimensional multi-omics datasets and model complex nonlinear relationships that may be difficult to capture with conventional linear approaches [120,122]. Supervised machine-learning approaches can integrate genomic and other omics features to support candidate-gene prioritization and phenotype prediction in plants [108,122]. In maize, integration of genomic, phenomic, and metabolomic data with machine-learning models improved grain-yield prediction, with a Random Forest model increasing prediction accuracy from 0.32 to 0.43 when multiple omics layers were combined [123]. Deep learning models applied to multi-environment SNP data have improved flowering-time prediction in maize beyond traditional Bayesian approaches, with the largest gains coming from multi-trait and multi-environment modeling [124]. The general architecture by which machine learning integrates multiple omics layers for GWASs is shown in Figure 5.

Despite this progress, integrating heterogeneous omics datasets remains technically demanding, and most multi-omics studies are still conducted under controlled conditions that may not represent field performance [108]. Ongoing priorities include single-cell multi-omics, pangenome-based GWASs that capture presence/absence variation across diverse genotypes, and tighter coupling with CRISPR-based validation and speed breeding to accelerate trait discovery and deployment in cereal crops [90,125,126,127].

## 7. Translational Applications: Turning GWASs into Breeding Impact

Representative recent studies illustrate how these elements come together across the four cereals. In wheat (*Triticum aestivum*), *TaMYB7-A1* was prioritized by integrating GWASs, eQTL mapping, population transcriptomics, and SMR, followed by functional validation in EMS mutants and transgenic lines [46]. In barley (*Hordeum vulgare*), pangenome analysis of wild and domesticated accessions revealed extensive structural variation at complex loci [47], while independent candidate-gene resequencing linked variation in *HvMADS56* to lateral spikelet morphology [128]. In maize (*Zea mays*), pangenome resources built from drought-contrasting germplasm have identified candidate genes for drought resistance [48]. In rice (*Oryza sativa*), large-scale meta-analysis across resequencing panels and graph-based pangenome references has increased discovery power and gene-mining efficiency [49]. Together, these studies show where cereal GWASs are heading: larger cross-study integration, explicit treatment of structural and gene-content variation, and tighter coupling between association signals and functional follow-up. The value of these discoveries, however, depends on translation into breeding decisions, as demonstrated by the release of improved wheat lines derived from an AB–NAMIC population designed to identify and accumulate beneficial alleles [129]. For cultivar development, GWAS outputs should therefore be evaluated in terms of which parents to cross, which lines to advance, and which alleles to edit or introgress, ideally considering haplotypes, allele frequencies in breeding pools, effect sizes across environments, and compatibility with selection schemes [110].

### 7.1. Marker-Assisted Breeding

Markers derived from robust GWAS loci can support marker-assisted selection (MAS) for large-effect traits, particularly disease resistance and some quality traits. For polygenic traits, haplotype-based selection offers an intermediate strategy: defining favorable haplotypes at several loci and selecting for haplotype combinations (trait stacking) [130]. For translational breeding, favorable haplotypes should be evaluated in relevant breeding populations with respect to their frequencies, estimated effects, and stability across environments [110,130].

### 7.2. Integrating GWASs with Genomic Selection (GS)

Where marker- and haplotype-assisted selection target a few large-effect loci, genomic selection (GS) takes the complementary approach of using genome-wide markers to predict breeding values and is particularly well suited to highly polygenic traits [131,132,133]. GWASs and GS are complementary: GWASs can identify major loci and inform feature selection, while GS captures diffuse polygenic background [134]. In bread wheat, a large-scale study combined GWAS across 50 traits with genomic prediction for 35 traits evaluated in international breeding environments, illustrating the joint use of association mapping and genomic selection within a breeding framework [135]. Hybrid models that include significant GWAS loci as fixed effects within GS can improve prediction when large-effect loci exist, and multi-trait GS can exploit correlations between traits [134,136]. In maize and wheat, the integration of stress-specific indices and multi-environment models is especially valuable for climate adaptation breeding [137,138].

### 7.3. Genome Editing and De Novo Allele Design

Beyond selecting among existing alleles, genome editing enables direct manipulation of GWAS-prioritized genes, offering a route to create or recreate favorable alleles, tune gene expression, or break linkage between beneficial and deleterious variants [91,139]. However, editing strategies should be guided by evidence of causality, pleiotropy assessment, and breeding context [110]. Pangenome resources help by clarifying which gene copies or alleles exist in which backgrounds [87]. Future pipelines may combine GWAS-derived candidate lists with AI-assisted prioritization and precision genome editing to accelerate genomics-assisted crop improvement [139,140]. Table 1 summarizes representative high-confidence GWAS hotspots and candidate genes across the four major cereals, together with the translational evidence supporting their use in breeding.

A validated allele is not yet a deployed one. Even once a candidate gene is confirmed, introgression of its favorable allele into elite, locally adapted backgrounds through conventional crossing can require repeated backcrossing, while linkage drag may retain deleterious donor-genome segments around the target locus [141,142]. Genome editing can shorten this timeline, but gene-edited crops face differing and evolving regulatory treatment across jurisdictions, which can affect pathways to commercialization [143]. Markers validated in one panel or environment do not always hold in another: effect sizes estimated under the population structure and environmental range of a discovery panel can shrink or disappear in a different genetic background or climate, so often, GWAS-derived markers still require local re-validation before routine use in a breeding program [36,70].

**Table 1:** GWAS-derived hotspot genes in the four major cereals (rice, maize, wheat, barley), combining representative hotspot loci with their primary breeding-objective category and translational evidence.

Crop	Locus/Candidate Gene(s)	Trait Category	Translational Evidence	Key References
Rice	*OsSPL13/GLW7* (Chr 7)	Yield/grain size	Increased grain length and yield; the large-grain allele in tropical japonica was introgressed from indica under artificial selection.	[144]
Rice	*GF14h* (Chr. 11)	Temperature-dependent seed germination	Natural 4 bp InDel identified by G×E GWAS and functionally validated; allele distribution suggests a role in geographical adaptation.	[145]
Maize	*ZmVPP1* (Chr. 9)	Abiotic stress/drought tolerance	GWAS-identified natural *ZmVPP1* promoter allele functionally validated in transgenic maize; potential target for selection and genetic engineering.	[146]
Maize	*ZmNAC111* (Chr. 10)	Drought tolerance/water-use efficiency	GWAS-identified 82 bp promoter MITE associated with drought tolerance; functional validation through transgenic maize confirmed the role of *ZmNAC111* expression.	[147]
Wheat	*TaGW8* (Chr. 7B)	Kernel size/thousand-grain weight	GWAS-identified *TaGW8-B1* locus from a 90K SNP panel; subsequent cloning revealed a 276 bp InDel, with the *TaGW8-B1a* allele associated with larger kernels and higher thousand-kernel weight.	[148]
Wheat	*TaFT-D1* (Chr. 7D)	Grain weight and grain size	GWAS-identified 1 bp InDel in *TaFT-D1* associated with grain weight; causal function confirmed by CRISPR-Cas9 knockout, with the favorable *TaFT-D1*(G) allele associated with higher thousand-grain weight.	[30]
Barley	*HvHKT1;5* (Chr. 4H)	Salinity tolerance/Na^+^ homeostasis	GWAS mapped salt-tolerance-associated SNPs to the *HvHKT1;5* region; ion-content and expression analyses supported its role in limiting Na^+^ accumulation in photosynthetic tissues.	[149]
Barley	*Int-c/HvTB1* (Chr. 4H)	Lateral spikelet fertility/spike architecture	Genome-wide and local association mapping, together with mutant analysis, identified *INT-C/HvTB1* as a modifier of lateral spikelet fertility; coding variants were associated with fertility phenotypes.	[150]

## 8. Applications in Crop Improvement

Having tracked a general pipeline from association to mechanism, the focus now turns to specific genes that have already completed that crossing. In cereal crops, a GWAS successfully resolved loci for agronomically important traits spanning yield components, phenology, plant architecture, stress tolerance, and nutritional quality [13]. Representative examples genuinely discovered through GWASs, rather than classical biparental mapping, include the rice grain-size regulator *OsSPL13/GLW7* and the maize drought-tolerance gene *ZmVPP1* (Table 1), both of which moved from a genome-wide association signal to a functionally validated, breeding-relevant allele [144,146]. Building on the methodological and resource advances detailed in the preceding sections, multi-locus and multi-environment models, pan-genomic references, multi-omics integration, and functional validation can be combined to strengthen the progression from association signals to biologically supported breeding targets [26,39,110]. Rather than re-cataloging individual reports, the following subsections organize GWAS applications by breeding objective, in turn examining disease resistance and biotic stress (Section 8.1), abiotic stress tolerance and climate resilience (Section 8.2), yield and quality (Section 8.3), and nutritional traits (Section 8.4). Across all four domains, the same translational logic applies: significant associations feed marker-assisted selection (MAS) for large-effect loci, while genome-wide marker data drive genomic selection (GS) for polygenic backgrounds, together shortening breeding cycles and increasing genetic gain [133,151].

### 8.1. Disease Resistance and Biotic Stress Tolerance

Biotic stresses caused by pathogenic fungi, bacteria, viruses, nematodes, and insect pests severely constrain cereal crop productivity and threaten global food security, with global yield losses associated with pathogens and pests estimated at 21.5% in wheat, 30.0% in rice, and 22.5% in maize [152]. GWASs provide a high-resolution route to the genetic architecture of resistance: by exploiting natural variation across diverse germplasms, it pinpoints resistance genes and quantitative trait loci (QTL) that can be deployed through marker-assisted selection (MAS) and genomic selection (GS), increasingly supported by transcriptomic and metabolomic evidence that clarifies the underlying defense pathways [44,153].

Several biotic-stress genes discovered specifically through GWASs have moved from statistical association to functional validation. In rice (*Oryza sativa*), the wall-associated kinase gene *Pb4* was identified through a GWAS of 249 accessions and it confers resistance to panicle blast; transgenic overexpression confirmed its protective effect [154]. In maize (*Zea mays*), a GWAS identified the F-box gene *ZmFBL41* as the causal gene for resistance to banded leaf and sheath blight, acting by protecting a lignin-biosynthesis enzyme from degradation [155]. These examples illustrate how GWASs, rather than classical biparental mapping, can identify functionally validated resistance loci with potential value for cereal breeding.

Modern studies increasingly integrate GWASs with multi-omics approaches, including transcriptomics, proteomics, and metabolomics, to strengthen candidate-gene prioritization and clarify molecular pathways involved in stress resistance [156]. Population structure and linkage disequilibrium can still generate false positives or obscure causal variants, and multi-locus models such as FarmCPU and BLINK, together with explicit modeling of genotype-by-environment interactions, are increasingly used to identify robust, context-dependent resistance loci [36,157].

### 8.2. Abiotic Stress Tolerance and Climate Resilience

Abiotic stresses such as drought, salinity, extreme temperatures, flooding, and nutrient deficiency impair cereal productivity and grain quality worldwide, and climate change is increasing their frequency and severity [158]. GWASs, combined with high-throughput phenotyping and multi-omics data, have accelerated the identification of genes and QTLs conferring tolerance to these stresses [108]. Exploiting functional diversity and predictive breeding approaches can further support the development of climate-resilient cultivars [159,160].

Drought tolerance is a major target in wheat improvement [161]. In maize (*Zea mays*), the vacuolar H+-pyrophosphatase gene *ZmVPP1*, first identified through a GWAS, has been functionally validated and represents a potential target for selection and genetic engineering for drought tolerance [146]. In rice, the QTLs qDTY1.1 and qDTY3.2 confer clear yield advantages under drought and have been deployed through marker-assisted breeding [162]. In barley, GWASs of a globally diverse spring barley panel under drought stress identified 79 significant SNPs and 35 high-confidence candidate genes associated with germination and seedling growth traits [163]. In wheat (*Triticum aestivum*), a GWAS of an association panel identified the zinc-finger transcription factor gene *TaDT1-A*, in which a promoter deletion enhances autophagy-mediated stomatal regulation and improves drought tolerance without a yield penalty, making it a direct target for marker-assisted selection [164].

Salinity tolerance provides a further example of this progression. In barley (*Hordeum vulgare*), a GWAS of a large mini-core collection mapped significant markers to the ion-transporter gene *HvHKT1;5*, and expression analysis confirmed its role in restricting sodium transport from root to shoot, supporting its use as a marker for salinity breeding [149]. A separate GWAS of 208 intermedium-spike barley accessions identified 38 significantly associated SNPs for germination and seedling traits under salinity stress, further illustrating the genetic diversity available for improving early-stage salt tolerance in barley [165]. Introgression lines and the nested-association Halle Exotic Barley-25 (HEB-25) population have identified and verified exotic wild-barley alleles and QTLs associated with improved salinity tolerance at germination and seedling stages [166].

Environmental variability, genotype-by-environment interactions, and the polygenic nature of stress tolerance continue to complicate the identification of strong markers for abiotic stress [37,108]. Genomic prediction and machine learning are increasingly used to model these complex trait architectures, and integrating GWASs with high-throughput phenotyping and omics validation is expected to accelerate delivery of climate-resilient cereal varieties [33,108,132,167].

### 8.3. Yield and Quality Traits

Yield improvement remains the primary objective of crop breeding, yet yield is a complex polygenic trait influenced by numerous genes with small effects and substantial environmental interactions [168,169]. GWAS has identified multiple QTLs for yield components, including grain number, grain weight and plant architecture across major crops [170,171]. The challenge lies in pyramiding favorable alleles while maintaining genetic balance and avoiding yield-quality trade-off [172,173].

Pan-genome-based GWASs have also revealed novel yield-related loci that single reference analyses overlook [49]. As described above (Section 4), a graph-based pan-genome meta-GWAS in rice recovered grain-size QTLs that were subsequently validated [49]. Quality traits, including grain nutritional composition, oil content, and dietary-fiber traits, have also been identified via GWASs [111,174,175]. In finger millet, a GWAS of 190 genotypes discovered genetic regions affecting grain nutritional content, including Fe, Zn, Mg, Ca, and protein levels [175]. Grain protein content in wheat has similarly benefited from GWASs: a genome-wide scan identified stable genetic loci and candidate genes controlling grain protein content across environments [176].

Metabolite GWASs have proven particularly valuable for quality traits [118]. The integration of metabolomics with GWASs enables a direct connection between genetic variants and metabolite profiles, facilitating biofortification efforts to develop nutritionally enhanced crops [2]. This approach has been successfully applied to identify candidate genes associated with specific metabolite profiles in multiple crop species [118,177].

### 8.4. Nutritional Traits

Nutritional improvement is a vital component of global food security, particularly in regions affected by malnutrition and micronutrient deficiency [178,179]. GWASs have contributed to resolving the genetic basis of nutritionally important grain traits, including protein and micronutrient accumulation in cereals [180]. Notably, some major nutritional loci discussed below, including *Gpc-B1* in wheat and *opaque2* in maize, were originally characterized through classical genetic approaches, with subsequent association studies helping to evaluate their allelic effects across diverse germplasm [181,182]. The clearest translational success is the wheat (*Triticum aestivum*) *NAC* transcription factor gene *Gpc-B1* (*NAM-B1*), which accelerates senescence and nutrient remobilization to the grain: functional markers for this locus are already used in breeding programs to raise grain protein, iron, and zinc content [182,183]. In rice (*Oryza sativa*), *OsAAP6* is a functionally characterized regulator of grain protein content, while GWAS has identified grain Fe and Zn associations near metal-homeostasis genes including *OsNAS1*, supporting their potential use in biofortification [184,185,186].

**Figure 6:**
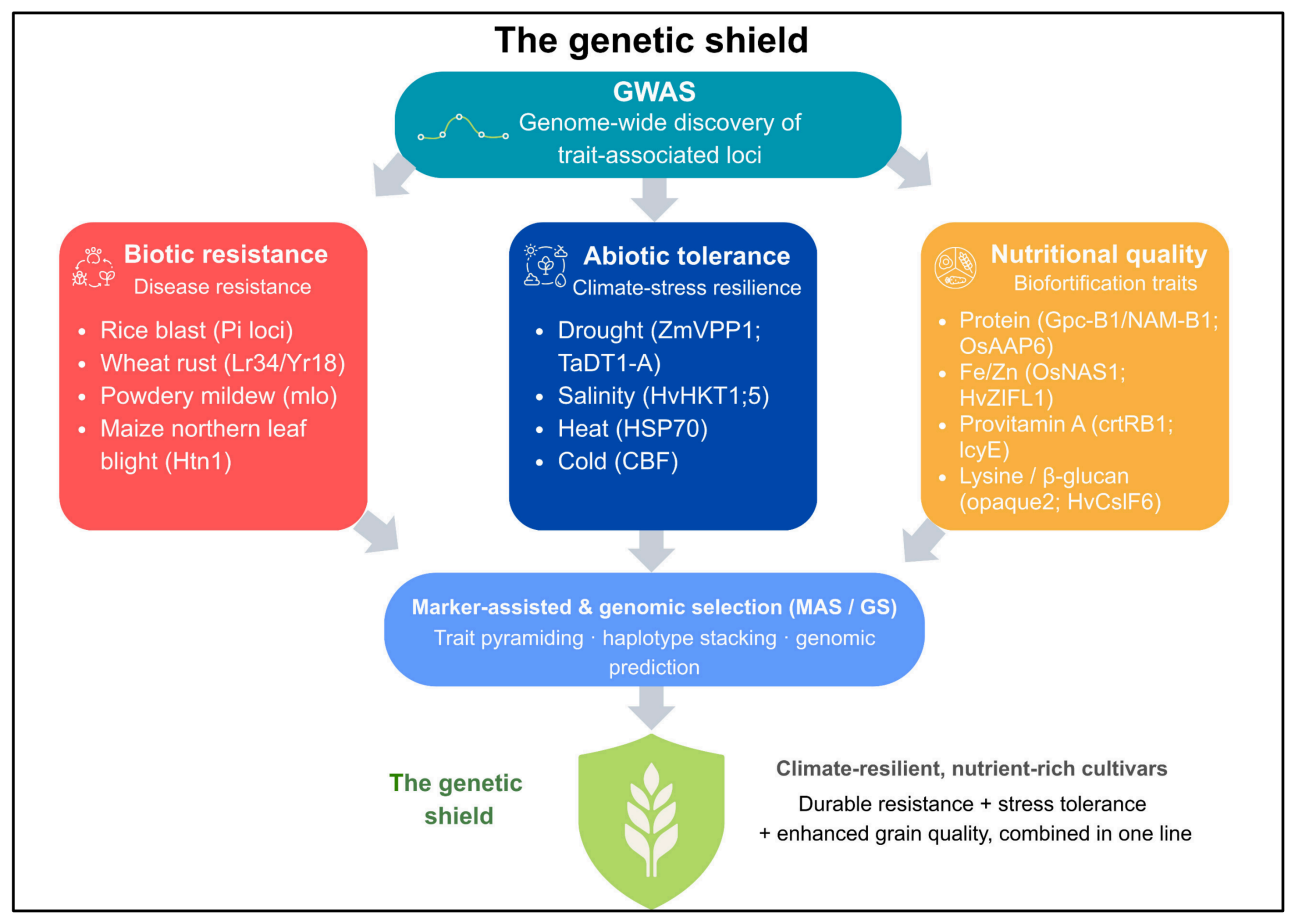
The genetic shield: using GWASs to build resilient cereal crops. Conceptual overview of how GWAS-derived loci for biotic resistance, abiotic stress tolerance, and nutritional quality are combined through marker-assisted and genomic selection to develop climate-resilient, nutrient-rich cultivars.

In maize (*Zea mays*), association analysis has linked variation at *opaque2* to kernel quality traits related to lysine content [181], while multi-environment GWAS has identified additional loci for protein, oil, starch, and lysine content [187]. Carotenoid GWAS has also confirmed *crtRB1* and *lcyE* as major loci relevant to provitamin A biofortification [188]. In barley (*Hordeum vulgare*), GWASs have linked variation in the *HvCslF6* region to grain β-glucan content [111], while a recent GWAS identified *HvZIFL1* as a strong candidate underlying variation in grain zinc concentration and developed diagnostic markers with potential value for biofortification breeding [189]. Collectively, the resistance, tolerance, and quality loci discussed across Section 8.1, Section 8.2, Section 8.3 and Section 8.4 can be viewed as a genetic toolkit for building resilient cereals (Figure 6).

## 9. Challenges, Limitations, and Future Perspectives

Climate change is accelerating the need for breeding methods that outperform traditional approaches, and GWASs remain central to that effort, but several persistent limitations constrain how quickly their discoveries translate into deployed cultivars [41,190]. One of the foremost limitations is the confounding effect of population structure and relatedness in association panels, which can lead to spurious associations or loss of statistical power [191]. Although mixed linear models (MLMs) and other statistical corrections have been developed, balancing false discovery rates and detection power in highly structured populations remains challenging [192].

Because many important agronomic traits are highly polygenic, individual GWAS loci often explain only a small proportion of phenotypic variance; genomic selection therefore provides a complementary strategy by using genome-wide marker effects [133,134]. Another constraint is that GWAS associations do not by themselves establish biological causality, and candidate genes therefore require functional follow-up through approaches such as reverse genetics, transcriptomics, or genome editing [193]. The validation gap can be narrowed by integrating multi-omics evidence with targeted genome editing and field-relevant validation to test whether prioritized GWAS candidates produce reproducible phenotypic effects [119].

A more understated problem is overcorrection: while mixed models account for population structure, aggressive correction may mask true associations when causal variants are themselves correlated with population structure as a result of selection history [194]. Striking the right balance between controlling false positives and preserving detection power, therefore, requires careful attention to population composition and statistical methodology [195]. High-resolution phenotyping remains another bottleneck. Traditional phenotyping approaches are labor-intensive, subjective, and often inconsistent across environments [33]. Advances in high-throughput phenotyping (HTP) platforms, such as unmanned aerial vehicles (UAVs), hyperspectral imaging, and automated greenhouse systems, have started to alleviate these limitations [196]. However, integrating high-quality phenotypic data with genotypic information remains a major challenge in cereal breeding [33].

Another critical issue is the limited ability of conventional GWASs to detect rare variants [35]. In addition, complex interactions among genes (epistasis) or gene-by-environment interactions may be missed in single-locus GWAS frameworks, leading to incomplete models of trait architecture [197].

Linkage disequilibrium complicates the identification of causal variants within associated regions [198]. Multiple variants in high LD may show similar association signals, making it difficult to determine which variant is functionally responsible for the phenotypic effect [26]. High-resolution genomic mapping, multi-omics integration, and functional validation through genome editing can help resolve causal variation, although their broad implementation remains technically and computationally demanding [199].

Genotype-by-environment interactions present both biological and analytical challenges for GWAS [38]. Phenotypic expression of many traits, particularly those related to stress tolerance and nutritional quality, is strongly influenced by environmental conditions [200,201]. Multi-environment trials combined with explicit G×E interaction models can improve the robustness and transferability of GWAS associations across environments, although these approaches require adequate sample sizes and computational resources [37,38]. As pangenome datasets continue to expand, computational scalability remains a practical challenge, requiring more efficient algorithms, standardized pipelines, and scalable data-storage strategies [83].

Translation into elite breeding pools carries its own practical barriers: incorporating a validated allele through conventional crossing takes multiple backcross generations, and genome editing shortens this timeline but faces uneven regulatory treatment across jurisdictions, as detailed earlier in the review [142,202]. Future progress will depend on closer integration of GWASs with multi-omics and expanding pangenome resources, together with rigorous validation of candidate loci in relevant breeding populations and target environments.

## 10. Conclusions

GWAS has moved beyond the detection of marker–trait associations and is increasingly used to connect genetic variation with gene function and breeding value. In cereal crops, pangenomes, improved statistical models, multi-omics integration, and functional validation are helping to distinguish well-supported candidate genes from statistical signals alone. The real impact of these advances will depend on whether validated loci remain effective across genetic backgrounds and environments and can be translated into practical selection, genomic prediction, introgression, or genome editing. Linking discovery, validation, and breeding application in a single workflow will therefore be central to turning GWAS results into more resilient and productive cereal cultivars.

## Data Availability

No new data were created or analyzed in this study. Data sharing does not apply to this article.
